# Conducting Electrospun Nanofibres: Monitoring of Iodine Doping of P3HT through Infrared (IRAV) and Raman (RaAV) Polaron Spectroscopic Features

**DOI:** 10.3390/nano12234308

**Published:** 2022-12-04

**Authors:** Alessia Arrigoni, Luigi Brambilla, Chiara Castiglioni, Chiara Bertarelli

**Affiliations:** 1 Dipartimento di Chimica, Materiali e Ingegneria Chimica Giulio Natta, Politecnico di Milano, Piazza Leonardo da Vinci 32, 20133 Milan, Italy; 2 Center for Nano Science and Technology @PoliMi, Istituto Italiano di Tecnologia, Via Pascoli 70/3, 20133 Milan, Italy

**Keywords:** regioregular poly(3-hexylthiophene-2,5-diyl), conducting polymers, molecular orientation, IR band polarisation, effective conjugation coordinate (ECC)

## Abstract

Aligned polymer nanofibres are prepared by means of the electrospinning of a chlorobenzene solution containing regioregular poly(3-hexyltiophene-2,5-diyl), P3HT, and poly(ethylene oxide), PEO. The PEO scaffold is easily dissolved with acetonitrile, leaving pure P3HT fibres, which do not show structural modification. Polymer fibres, either with or without the PEO supporting polymer, are effectively doped by exposure to iodine vapours. Doping is monitored following the changes in the doping-induced vibrational bands (IRAVs) observed in the infrared spectra and by means of Raman spectroscopy. Molecular orientation inside the fibres has been assessed by means of IR experiments in polarised light, clearly demonstrating that electrospinning induces the orientation of the polymer chains along the fibre axis as well as of the defects introduced by doping. This work illustrates a case study that contributes to the fundamental knowledge of the vibrational properties of the doping-induced defects—charged polarons—of P3HT. Moreover, it provides experimental protocols for a thorough spectroscopic characterisation of the P3HT nanofibres, and of doped conjugated polymers in general, opening the way for the control of the material structure when the doped polymer is confined in a one-dimensional architecture.

## 1. Introduction

The class of π-conjugated polymers exhibit intrinsically semiconducting and conducting behaviour owing to their low energy gap [1]. Moreover, both the energy gap and the frontier electronic levels can be tuned by chemical substitution of the monomeric units with electroactive substituents and using electron-rich or electron-poor monomeric units. This allows for a fine control over both the energy gap and the frontier energy levels, the latter being extremely relevant for charge injection and interface with electrodes. Moreover, doping provides a transition from semiconducting to conducting properties by the introduction of polaron and bipolaron defects through chemical redox or electrochemical processes [1,2,3,4]. Due to their peculiarities, π-conjugated polymers find applications in their pristine state, for example, in organic solar cells, light-emitting diodes, and field effect transistors, while in their doped state they are employed as artificial muscles, chemical and biosensors, and for organic thermoelectrics [5,6,7,8,9,10,11].

Though a huge variety of polymers and copolymers have been finely designed and synthesised to meet the requirements for performant electronic and optoelectronic devices, the regioregular poly(3-hexylthiophene-2,5-diyl) (P3HT) is one of the most widely studied, resulting in a great improvement in knowledge on the relationship between structure, processing and postprocessing, and charge transport [6,12,13,14,15,16,17]. The possibility to process these polymers into micro- and nanofibres opens up new opportunities related to the strong molecular, mechanical, and electrical anisotropy along the fibre axis, as well as the size confinement. Fibres exploit peculiar morphological features, which may affect the properties of the polymer sensitive to the molecular packing and orientation [18,19,20,21,22]. Polymer orientation along the fibre axis may have a significant effect on the charge transport mechanism [19,23], which mainly exploits intrachain hopping along the backbone in the fibre direction, while requires interchain hopping in the π–π stacking direction, mainly orthogonal to the fibre axis. Moreover, a downsized scale allows for the miniaturisation of devices, such as single-fibre organic field effect transistors [24]. Additionally, the possibility of obtaining fibrous membranes has led to the development of sensors where the response of the material to an external stimulus (such as the exposure to organic vapour or solutions) is large and fast due to the larger active surface area with respect to traditional compact sensing layers [8,19,23].

Electrospinning is a versatile and easy method to process conjugated polymers into fibres. Due to their intrinsically rigid backbones, poor solubility, and relatively low molecular weights, the electrospinning of semiconducting polymers is usually not straightforward since the critical numbers of chain entanglements required to form continuous fibres is not usually reached. [12,23,25,26]. An exception is provided by poly-para-phenylenevinylenes (PPVs), whose relatively flexible chains and high molecular weight allow for easy collection of fluorescent defect-free fibres [27]. Over the years, different strategies have been applied to overcome the issues related to the electrospinning of π-conjugated polymers: the surface polymerisation of the monomers onto electrospun polymer fibres [28,29,30], the coaxial electrospinning of the conjugated polymer with an outer spinnable polymer or solvent feed [12,13,16,31,32]**,** or the mixing of the conjugated polymer with a spinnable polymer in the starting solution, to assist the formation of fibres [18,33,34].

In this study, we prepared P3HT fibres blended with high-molecular-weight polyethylene oxide (PEO) to support fibre formation by electrospinning. PEO has been selected due to its ease of processability and its solubility in many solvents [18,24], in particular, in chlorobenzene, which is also a suitable solvent for P3HT and conjugated polymers, in general. Moreover, PEO is easily removed from the spun fibres by rinsing with selective solvents that guarantee its complete dissolution without any solubilisation of P3HT [18,23]. The rationale behind the PEO rinsing is based on the removal of the insulating matter, whose presence can have strong effects on the supramolecular organisation of the conjugated polymers, and hence on charge mobility [23]. The molecular structure of P3HT fibres and the effects of the presence of the PEO scaffold are investigated in this work by means of vibrational infrared and Raman spectroscopies. We also demonstrate the remarkable orientation of the polymer chains, of both the supporting PEO and the semiconducting P3HT, almost along the fibre axis, by means of IR measurements in polarised light.

Finally, doping of P3HT was performed by exposing the fibres to iodine vapours, which effectively occurred with and without the presence of the insulating PEO. Infrared investigation highlighted very strong doping-induced IRAV features assigned to vibrational modes of the doping-induced polaron defects. We also set up a method for estimating the doping degree, which enables the monitoring of doping/de-doping trends over time. Worth noting is that IR spectroscopy in polarised light clearly proves that IRAVs in doped samples also show a parallel polarisation, as already pointed out by reference [35].

Our fundamental study presents a method to monitor and characterise the doping of conducting polymers, highlighting, for the first time, the peculiarities related to the confinement into a fibrous 1D architecture. For this reason, we have selected a widely studied conjugated polymer, namely P3HT, and a simple and clean doping procedure (exposure to I_2_ vapours). The outcomes of the analysis here illustrated represent a test bed for future works, and, in particular, provide protocols, mainly based on spectroscopy data, for monitoring any doping process.

## 2. Materials and Methods

### 2.1. Materials

Regioregular poly(3-hexylthiophene-2,5-diyl) P3HT (Mw = 50,000–75,000 g mol^−1^ polydispersity index < 6.0, RR ≥ 90%, poly(ethylene oxide) PEO (Mw = 4,000,000 g mol^−1^), iodine, chlorobenzene, and acetonitrile were purchased from Sigma Aldrich (Schnelldorf, Germany). All chemicals were used as received without further purifications.

Polymeric solutions were prepared by dissolving the two polymers in a 70:30 P3HT:PEO ratio, for a total amount of polymer of 5% *w*/*w* in chlorobenzene (CB), according to previous studies [24]. Solutions were left under magnetic stirring and heating (at 40 °C) for at least 24 h, followed by 30 min of sonication before electrospinning. For the preparation of the reference fibres of PEO, a solution was prepared with 1% *w*/*w* polymer in chloroform; the solution was stirred at room temperature for 12 h until a clear solution was obtained.

### 2.2. Electrospun Fibres

Fibre samples were prepared by electrospinning using a horizontal electrospinning setup using a Spellman Sl-150 high-voltage power supply (Pulborough, West Sussex, UK). The feed solutions were loaded into a 1 mL glass syringe (Hamilton Gastight, model 1002 TLL) with a stainless steel needle (inner diameter = 22 gauge). The syringe was mounted on a mobile infusion pump (KDS Scientific, model series 200), to set the flow rate. P3HT:PEO fibres were collected in an aligned fashion onto a cylindrical rotating collector. Samples were electrospun using the following parameters: needle-to-collector distance of 22 cm, applied voltage of 13 kV, and solution flow rate of 0.5 mL h^−1^. To remove PEO, the electrospun fibres were washed with acetonitrile, leaving intact fibres of P3HT.

The reference PEO fibres were prepared by processing the PEO solution using the following parameters: needle-to-collector distance of 22 cm, applied voltage of 20 kV, and solution flow rate of 0.4 mL h^−1^.

### 2.3. Doping

Both P3HT:PEO and P3HT fibres were doped by exposing them to iodine vapours by inserting the samples for 1 min into a closed chamber containing iodine crystals.

### 2.4. Characterisation

#### 2.4.1. FT-IR Spectroscopy

The IR absorption spectra were recorded using a Nicolet NEXUS FT-IR spectrometer (4 cm^−1^ resolution, 128 scans) equipped with a Thermo-Electron Corporation Continuum FT-IR microscope. Spectra of nanofibres collected onto silicon substrate were recorded at room temperature in transmission mode. A ZnSe wire grid IR polariser was used with the same setup for dichroism analysis of aligned nanofibres.

#### 2.4.2. FT-Raman Spectroscopy

Raman spectra of pristine and doped nanofibres were recorded with a Nicolet NXR9600 FT-Raman spectrometer using a Thermo Scientific MicroStage FT-Raman Microscope. Spectra were collected in backscattering geometry focusing on fibres mat deposited on aluminium foil (1064 nm exciting laser line, 100 mW, 512 scans at 4 cm^−1^ resolution).

#### 2.4.3. Scanning Electron Microscopy

SEM images were taken by means of a Tescan scanning electron microscope (Tescan, MIRA3, Brno, The Czech Republic). The accelerating voltage applied for imaging was 3 kV. Fibres were directly deposited onto a silicon substrate, and no metallisation was needed. The measurements of the nanofibre diameters were carried out by processing SEM images with the software ImageJ (Rasband, W.S., ImageJ, U.S. National Institutes of Health, Bethesda, MD, USA, 1997–2016). The statistical analysis of diameter distribution was carried out with Origin software (ver. 2021), considering 100 independent measurements of fibres diameters for each sample.

## 3. Results and Discussion

This section reports the results of the analysis carried out for the structural characterisation of the following samples:

1. P3HT:PEO in the pristine (spun) and in the iodine-doped form;
2. P3HT fibres after PEO removal, in the pristine and in the iodine-doped form;
3. PEO fibres, spun.

### 3.1. Scanning Electron Microscopy: Fibre Morphology

The parameters adopted in the electrospinning process determine the quality and the structure of the fibres. These parameters are often cross-related, resulting in a highly controllable process [36,37]. In this work, we started from previously developed process parameters, while we re-optimised the value of the applied voltage. Together with polymer concentration, the average polymer molecular mass played a relevant role in reaching the critical number of entanglements for fibre formation. Indeed, when P3HT molecular mass is too low, only fibres with an irregular morphology are collected at any experimental condition tested (see Supplementary Materials). Moreover, in this case, rinsing of PEO with acetonitrile leads to a significant change in fibre morphology, with fibres breaking into short segments (see Figure S1).

**Figure 1:**
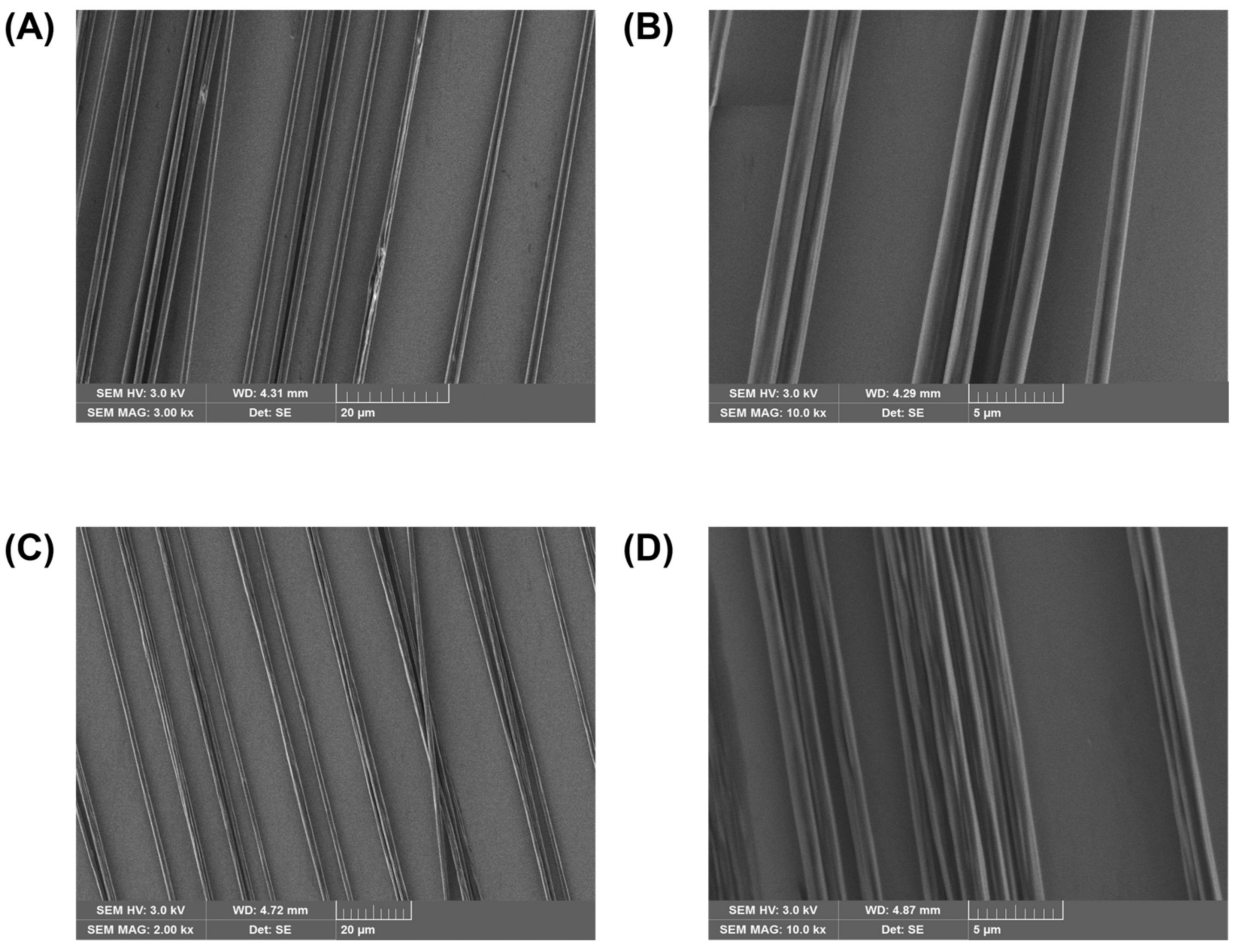
SEM micrographs of P3HT:PEO nanofibres: panels (**A**,**B**) as deposited; panels (**C**,**D**) P3HT samples after removal of PEO.

P3HT:PEO defect-free, smooth, and continuous fibres were collected using P3HT with a higher molecular mass (Mw) of 50,000–75,000 g mol^−1^, thus indicating a good optimisation of the set parameters and the stability of the process (see Figure 1A,B). No changes in fibre shape and surface appearance resulted from the rinsing in acetonitrile to remove the supporting PEO, as shown in Figure 1C,D. Only a reduction in the fibre size is noticed, as expected (Figure S2).

Interestingly, the diameter distribution is narrow for both samples, further confirming the stability of the electrospinning process (Figure S2). The macroscopic alignment of the fibres is remarkable, and it is not lost after the removal of PEO scaffold.

### 3.2. Vibrational Spectra

#### 3.2.1. The ECC Theory: Raman and IR Spectra of Polyconjugated Molecules

Since the early synthesis of organic semiconducting polymers and oligomers, Raman and infrared (IR) spectroscopies have proven their effectiveness for the study of their molecular and electronic structure [1,38,39,40]; furthermore, the strong electron–phonon interaction is ultimately responsible for several peculiar spectroscopic features. In particular, the Raman spectrum is an invaluable tool to unveil how delocalised π-electrons are affected by chemical substitutions, conformational changes, and by intra- and intermolecular interactions in crystalline or in disordered phases [41,42,43,44,45]. On the other hand, electronic processes, such as photoexcitation or chemical doping, often lead to a remarkable relaxation of the molecular geometry, which in turn determines the IR and Raman spectroscopic responses [42,46,47,48,49,50,51,52].

Starting from the first investigations [38,53,54,55,56,57,58,59], several spectroscopic studies on oligo- and polythiophenes (PT) have been published, dealing with detailed structural information of both unsubstituted and substituted molecular skeletons. Indeed, methods for suitable functionalisation of the thiophene rings (e.g., with alkyl chains), as well as control over the material morphology through structural features, such as molecular weight distribution and chain regioregularity, and over the processing phase, have been designed aiming at material optimisation in the perspective of applications in organic electronics, optoelectronics, sensing, and energy harvesting.

**Scheme 1:**
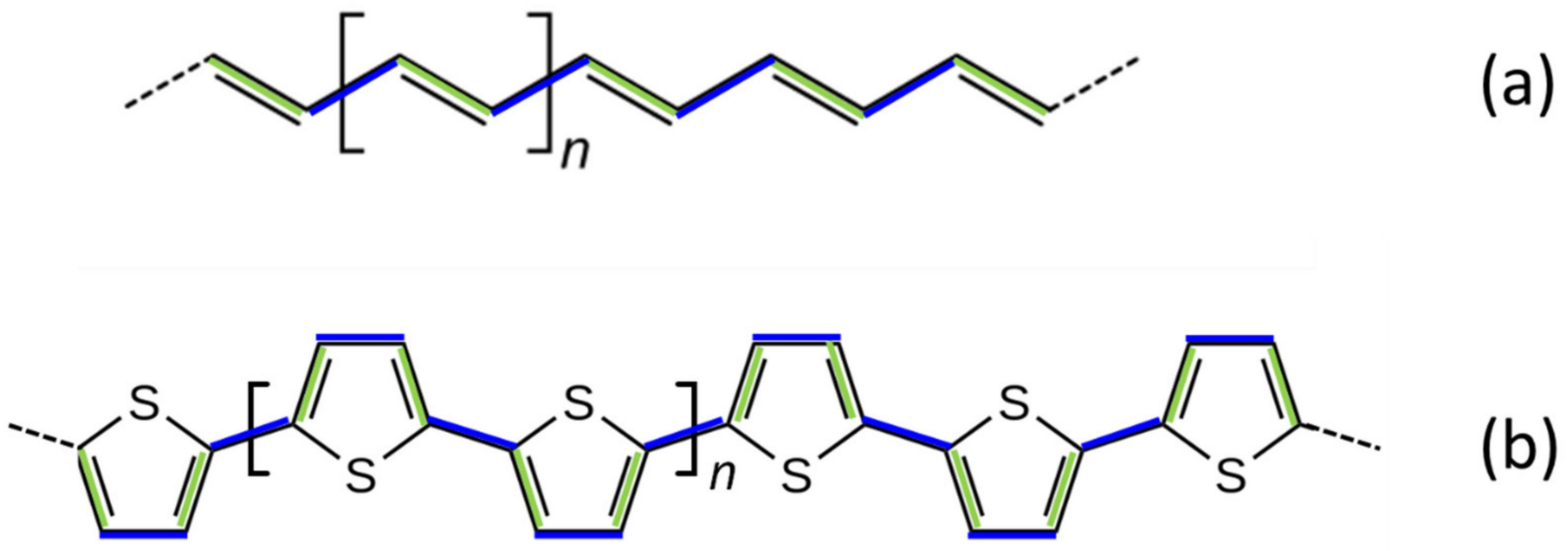
Schematic illustration of the vibrational displacements associated with the collective ECC coordinate (**a**) for polyacetylene and (**b**) for polythiophene. Coloured segments are associated with CC bonds, which stretch (green) and shrink (blue) simultaneously.

According to the effective conjugation coordinate (ECC) theory proposed by Zerbi et al. [42,60,61,62], the strongest Raman bands of linear conjugated polymers and oligomers are assigned to collective out-of-phase stretching vibrations of the conjugated C=C/C–C bonds along the molecular backbone, which are often referred to as ECC modes or Я modes. ECC vibrations cause the oscillation of the average bond length alternation (<BLA> = <R_C–C_ − R_C=C_>) [42,48]; they are usually coupled with CH wagging and, in some cases, with other vibrational displacements [41,42,48,51,55,63,64,65]. For this reason, more than one ECC band is often observed in the Raman spectrum: indeed, more than one vibrational normal mode (or phonon) can exhibit atomic vibrational displacements with a large projection along the ECC trajectory. Scheme 1 describes the changes in the CC bond lengths associated with the ECC vibration of a polyene chain and of a polythiophene chain.

The ECC theory, first developed for the study of linear polyene molecules and polyacetylene, allowed for the rationalisation of the remarkable downward shift of the two ECC bands, while increasing the chain length. Moreover, it predicts a remarkable modulation of the intensity pattern of the Raman spectrum, coming from the increase in the delocalisation of π-electrons while increasing the size of the molecule. The last feature is also observed in the case of polyconjugated chains based on aromatic or heteroaromatic units, such as polythiophenes [65]. In contrast, the frequency dispersion of the ECC modes with chain length is strongly dependent on the aromaticity of the constituent monomers, and polythiophenes usually do not exhibit this feature [66]. Raman frequencies and intensities of ECC bands are also affected by the presence of structural defects (e.g., conformational defects), because of their effect on the confinement and on the polarisation of π-electrons [41,42,43,44,45,46,65].

In addition to the Raman investigations, IR spectroscopy showed its great potential in the study of the electronic structure of push–pull polyconjugated molecules [44,45] and doped semiconducting polymers [42,46,47,49,50,51,67,68,69]. The spectroscopic analysis of push–pull structures is a milestone in the understanding of the spectroscopic response of polyconjugated molecules, since it demonstrated that the strong Raman active ECC modes can acquire a very large IR dipole transition if the π-electrons system is polarised. This occurs in push–pull polyenes, because of the presence of two end groups with electron-withdrawing and electron donor characteristics, respectively [44,45]. In a similar way, the charge transfer occurring upon chemical or electrochemical doping allows for the activation in the IR spectrum of vibrational transitions associated with ECC modes, often showing absorption intensities comparable to those ones of electronic transitions [42,46,47]. Doping-induced IR bands were initially referred to as IR active vibrations or IR activated vibrations (IRAVs) [48,49,51], and, more recently, they have been renamed as IR intense vibrational modes (IVMs) [50]. Though the phenomenon of the appearance of very strong IRAVs was first rationalised in the case of doped polyacetylene, it concerns several doped polyconjugated organic materials, and accompanies the remarkable increase in electric conductivity, earning them the name of “conducting polymers” [1,38,50]. The appearance of new, often very strong, vibrational transitions in the IR spectra of doped polymers is empirical evidence that a “charged defect” has been formed. Indeed, IRAV are often described as the vibrational signature of charged solitons, polarons, or bi-polarons, which are created on the polymer chains in different doping regimes. Raman evidence of the polaron/bi-polaron formation has also been reported [35,42,46,70,71,72,73]. Starting from different formalisms, both the amplitude mode [51] and the ECC theory [60] have identified the IRAVs as modes involving the BLA oscillations (i.e., ECC modes) of the charged defect in a doped chain. The remarkable downward frequency shift of the IR active ECC modes of a doped chain compared to the corresponding ECC Raman features of the neutral species is ascribed to the softening of the collective vibrational force constant associated with the ECC coordinate, or, according to the AM theory, it is described by the renormalisation of the vibrational frequencies upon charging. Moreover, the activation of the ECC modes in the IR spectrum of doped polyacetylene (i.e., the onset of IRAV) is justified by symmetry breaking induced by the charge transfer.

ECC theory and AM theory are based on a phenomenological approach and simplified physical models, which have been revisited recently on the light of state-of-the-art quantum chemical density functional theory (DFT) simulations on model systems of neutral and doped polyacetylene [74]. The analysis proves the robustness of the early interpretation, allows the evaluation of the large charge fluxes associated with ECC of a doped chain, and brings to light that ECC vibration controls phonon-assisted intramolecular charge hopping [74].

The application of the ECC theory to polymers more complex than polyacetylene, such as polythiophenes, is rather straightforward, and it explains the appearance of strong IRAVs while doping [44]. However, the increasing number of degrees of freedom in the vibrational space introduces some complexity and new features. Indeed, the coupling of ECC with several degrees of freedom increases the number of strong doping-induced IR features, and it often results in the appearance of a structured band, covering a wide wavenumber range. In addition, band broadening can arise because of the occurrence of a variety of the charge defects, whose size and structure are modulated by the intra- and intermolecular environment, e.g., by the presence of conformational defects in the polymer chain or by the presence of different, more-or-less ordered, solid-state phases.

This paper deals with the IRAV of P3HT, in the form of electrospun nanofibres. IRAVs of doped polythiophene and of several polyalkylthiophenes have been observed and discussed for decades [35,58,59,70,71,72,73,75,76,77,78], and they have recently been the objects of new studies, driven by the renewed interest in doped conducting polymers as promising candidates for organic thermoelectrics [23,79].

Because of the similarity of the backbone structure, PT and P3HT show similar spectral features, as expected based on the ECC theory. On the other hand, as pointed out in previous detailed spectroscopic works on pristine (neutral) P3HT, and on their oligomers [63,64], the presence of alkyl chains at the β-position of the thiophenes lowers the whole polymer symmetry and determines a peculiar vibrational dynamic. In particular, the coupling between the stretching of CC bonds belonging to the alkyl chains and that of CC bonds belonging to the thiophene ring modulate ECC normal modes, and it affects their frequencies and Raman/IR intensities [63,64]. Notably, the strongest Raman active ECC mode is also active in IR, because of the symmetry lowering caused by the alkyl chain [63,64].

**Figure 2:**
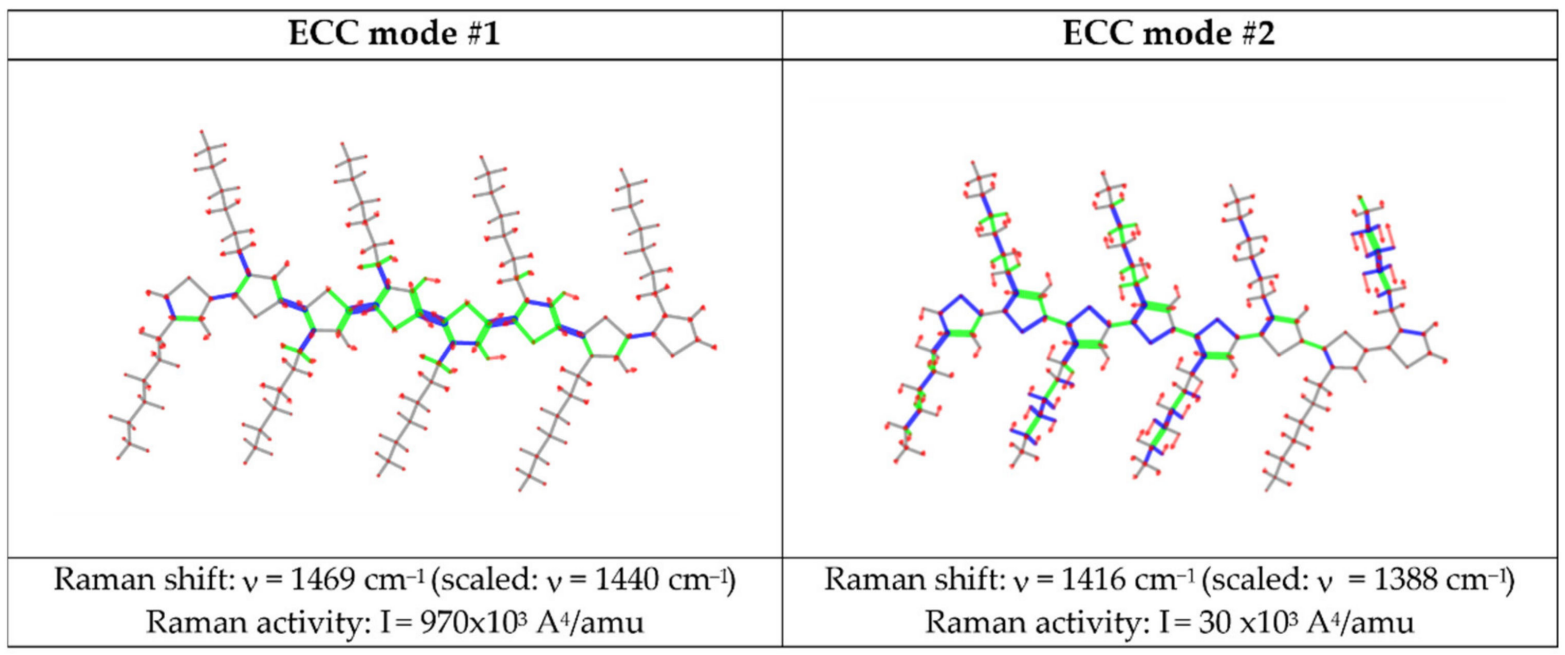
Computed vibrational displacements associated with the two strongest Raman bands of a 3H8T molecule. Calculations have been carried out with density functional theory simulations at the B3LYP/6-31G(d,p) level; details are in reference [63]. Red arrows represent displacement vectors; CC bonds are represented as green and blue lines of different thicknesses according to their relative stretching (shrinking).

Despite the complexity of its vibrational structure, the Raman spectrum of P3HT is still dominated by transitions associated with normal modes that involve ECC oscillation, as proven by DFT calculations applied to a model oligomer (3H8T), and illustrated in Figure 2. The CC stretching pattern of the ECC coordinate of Figure 1b is nicely followed by the vibrational eigenvector of ECC mode #1, depicted in Figure 2. However, it shows a smaller contribution from the stretching of the quasi-single CC bonds of the rings. A collective stretching of the quasi-single C–C bonds characterises ECC mode #2 (Figure 2), giving rise to a strong Raman transition, because of its non-vanishing projection on the ECC trajectory. From Figure 2, it is also evident that both the ECC modes involve CS stretching and bending of the CH bonds, due to their dynamic coupling with CC stretching.

The IR spectrum of a doped P3HT sample shows new, very strong bands, usually taken as the signature of the doping, i.e., of the formation of polarons. By analogy with doped PT, we assign these features to ECC modes involving the conjugated CC bonds sequence, which become polar after the electron transfer to the dopant.

Herein, we aimed at the study of the chemical doping of P3HT electrospun nanofibres, focusing in particular on:

- Whether the doping is effective both in washed fibres (pure P3HT fibres) and in the presence of the PEO scaffold;
- The monitoring of the de-doping over time via IR measurements;
- The analysis of the molecular orientation in fibres and consequent polarisation properties of the IRAV bands.

In this framework, the appearance of IRAV in the IR spectrum of P3HT will be taken as the signature of the charged defect, while we refer to the literature [55,70] for the detailed discussion of vibrational assignments of PT and P3HT doping-induced bands.

#### 3.2.2. IR Spectra of Pristine P3HT Fibres

**Figure 3:**
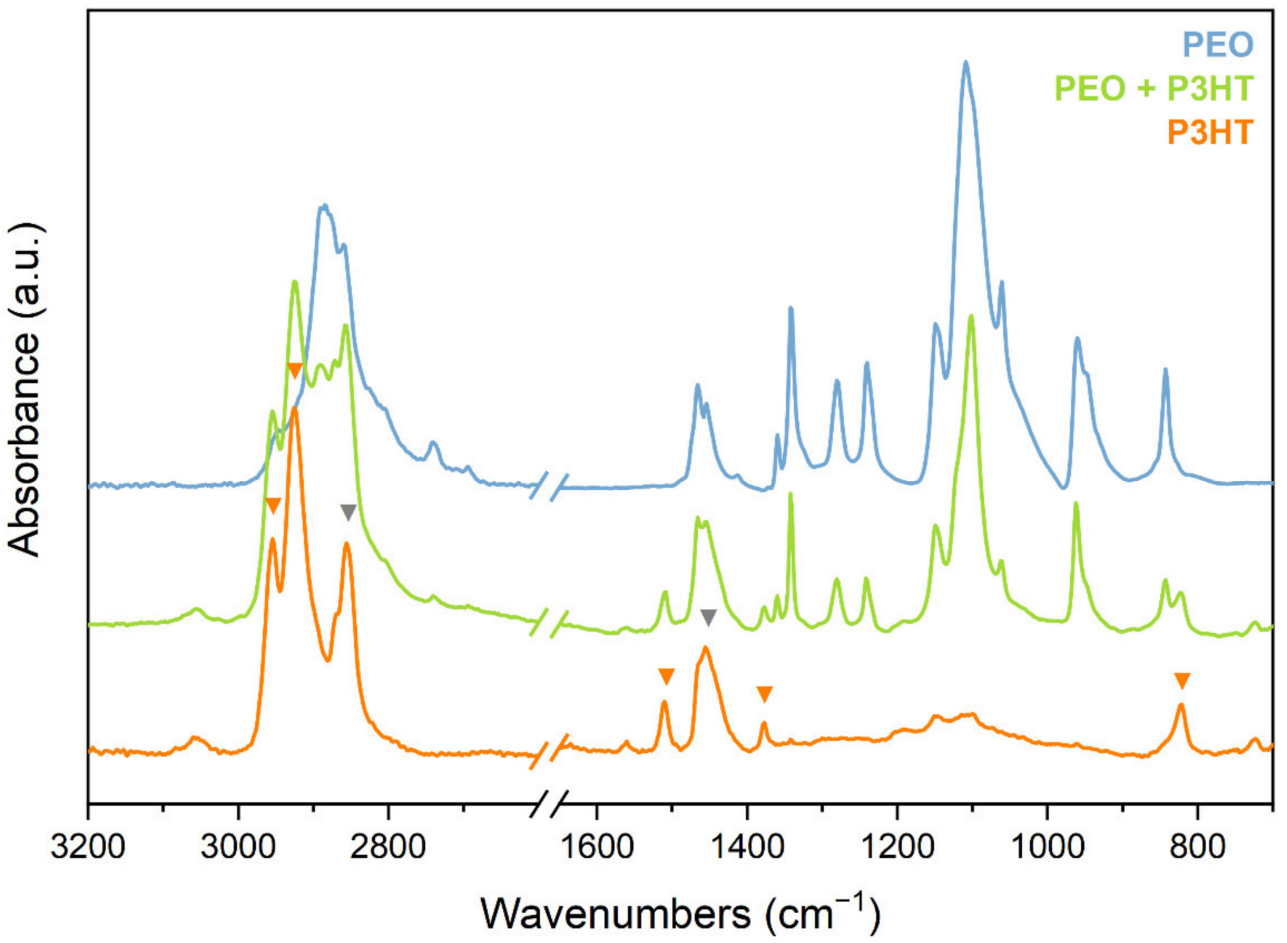
Infrared spectra of nanofibres, namely: pure PEO, **blue line**; P3HT:PEO, **green line**; P3HT (washed P3HT:PEO fibres), **orange line**. Spectra of P3HT and P3HT:PEO (orange and green line) have been normalised to CH stretching bands (2900 and 2955 cm^−1^) of P3HT, the PEO spectrum (blue line) has been normalised to the P3HT:PEO spectrum (green line), considering the 1342 cm^−1^ sharp peak of PEO. Orange triangles highlight features of P3HT that do not overlap with PEO peaks, and grey triangles indicate P3HT features showing a large overlap with PEO absorptions.

In Figure 3, the spectra of the pristine electrospun fibres obtained as (a) pure PEO, (b) P3HT:PEO blend, and (c) P3HT (after removal of PEO by washing a P3HT:PEO sample) are shown.

In the figure, some marker bands of P3HT are labelled from A to D (peak wavenumbers and their vibrational assignment are reported in Table 1. A, C, and D bands are located in spectral ranges free from IR transitions of PEO, such that the presence of P3HT in the blend (sample) can be immediately recognised. Both the absorption bands of PEO and P3HT in the spectrum of the blend do not show appreciable changes in peak wavenumbers and band shapes, thus suggesting that in the fibres, PEO and P3HT form a physical mixture, hence phase separation between the two polymers likely occurred by solvent evaporation during the electrospinning process. The last feature explains why, after washing, we obtained continuous and homogeneous fibres of P3HT (Figure 1).

**Figure 4:**
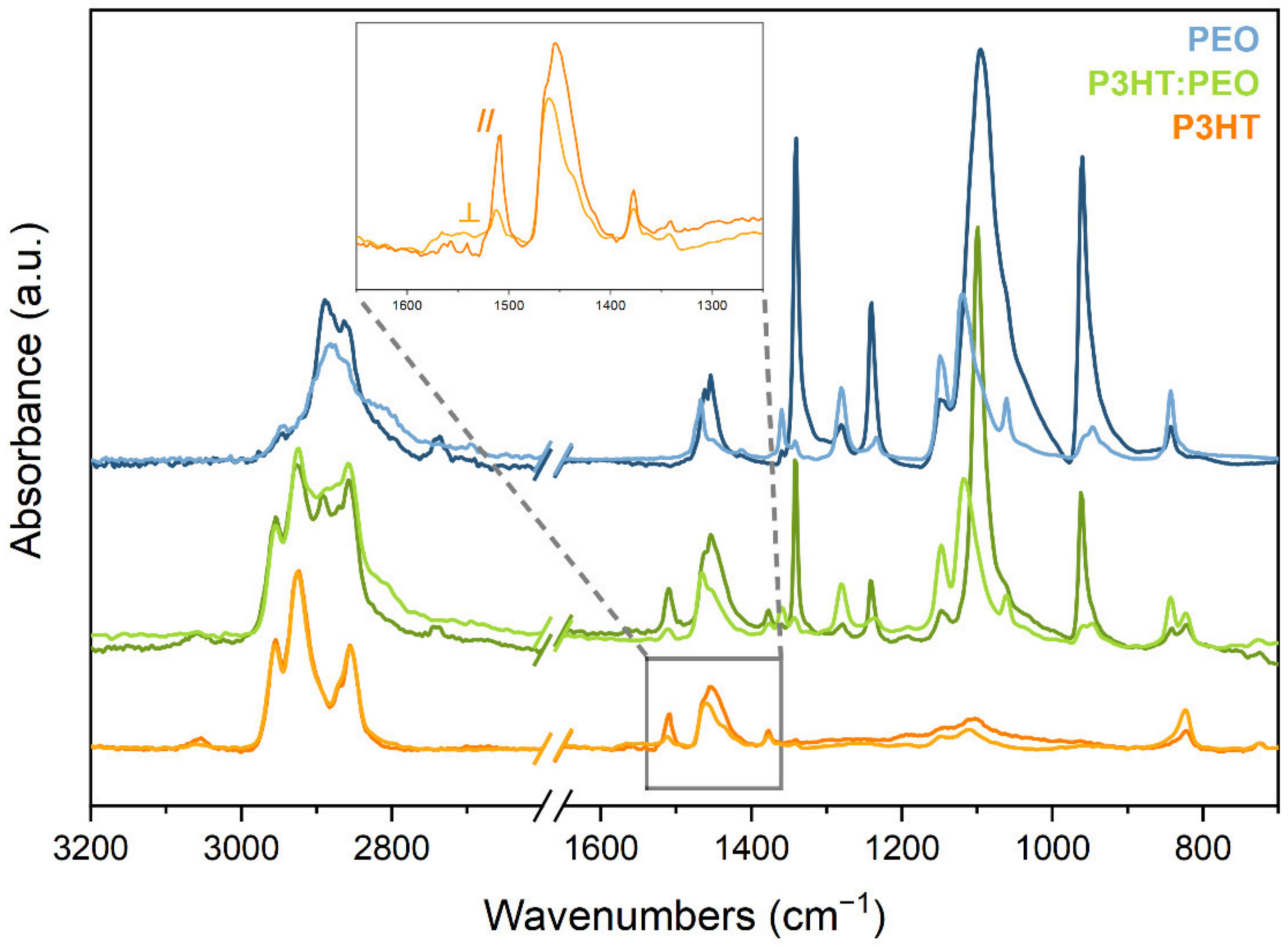
Infrared spectra of PEO (**blue lines**), P3HT:PEO (**green lines**) and P3HT (**orange lines**) macroscopically aligned nanofibres in linearly polarised light. Lighter coloured lines refer to spectra recorded with light polarised perpendicular to the nanofibre axis direction, while darker lines refer to spectra recorded with light polarised parallel to the nanofibres’ axis direction.

The collection of fibres onto a rotating drum allows one to obtain macroscopically oriented fibres, which in turn can be analysed by means of polarised IR spectra. In Figure 4, we report the IR spectra obtained with light linearly polarised in the fibre axis direction (// polarisation) and perpendicular to the fibre axis (⊥ polarisation) for PEO, P3HT:PEO, and P3HT samples.

All the spectra show evident dichroism, which confirms a remarkable degree of orientational order at the molecular level. The behaviour of pure PEO fibres parallels the observations reported in the literature for a uniaxially oriented crystalline sample of polyethylene glycol [80].

In particular, the quasi-total extinction of the CH_2_ rocking band at 961 cm^−1^ and of the CH_2_ wagging/twisting band at 1341 cm^−1^ in the perpendicularly polarised spectrum tells us that the polymer backbone is well aligned along the fibre axis. Interestingly, the same dichroic behaviour for the absorption bands of PEO is observed for P3HT:PEO blend, thus proving that the presence of a large amount of P3HT does not hinder the molecular orientation of PEO chains.

The assessment of the orientation of P3HT chains is not so trivial since P3HT molecules can form small crystal lamellae, of which orientation in a fibre is not easily predictable. For this reason, a careful analysis is necessary, considering the direction of the transition dipole of some specific marker bands. We focus on the A peak, assigned to a vibrational normal mode, Q_A_, which mainly involves a collective antisymmetric C=C stretching (R-) of the thiophene rings. Considering the local symmetry of a bare thiophene ring (alkyl chains are neglected), we can predict the direction of the dipole moment derivative ∂**M**/∂R- according to the irreducible representation of the B_1_ species: for B_1_ modes the only ∂M^x^/∂R- component is non-vanishing, *x* being the horizontal axis (orthogonal to the C_2_ vertical axis) in the plane of the ring. In a polythiophene chain, the *x*-axis of the individual rings is parallel to the chain axis, and we can infer that the vector ∂**M**/∂Q_A_ is directed along the polymer axis *x*.

**Figure 5:**
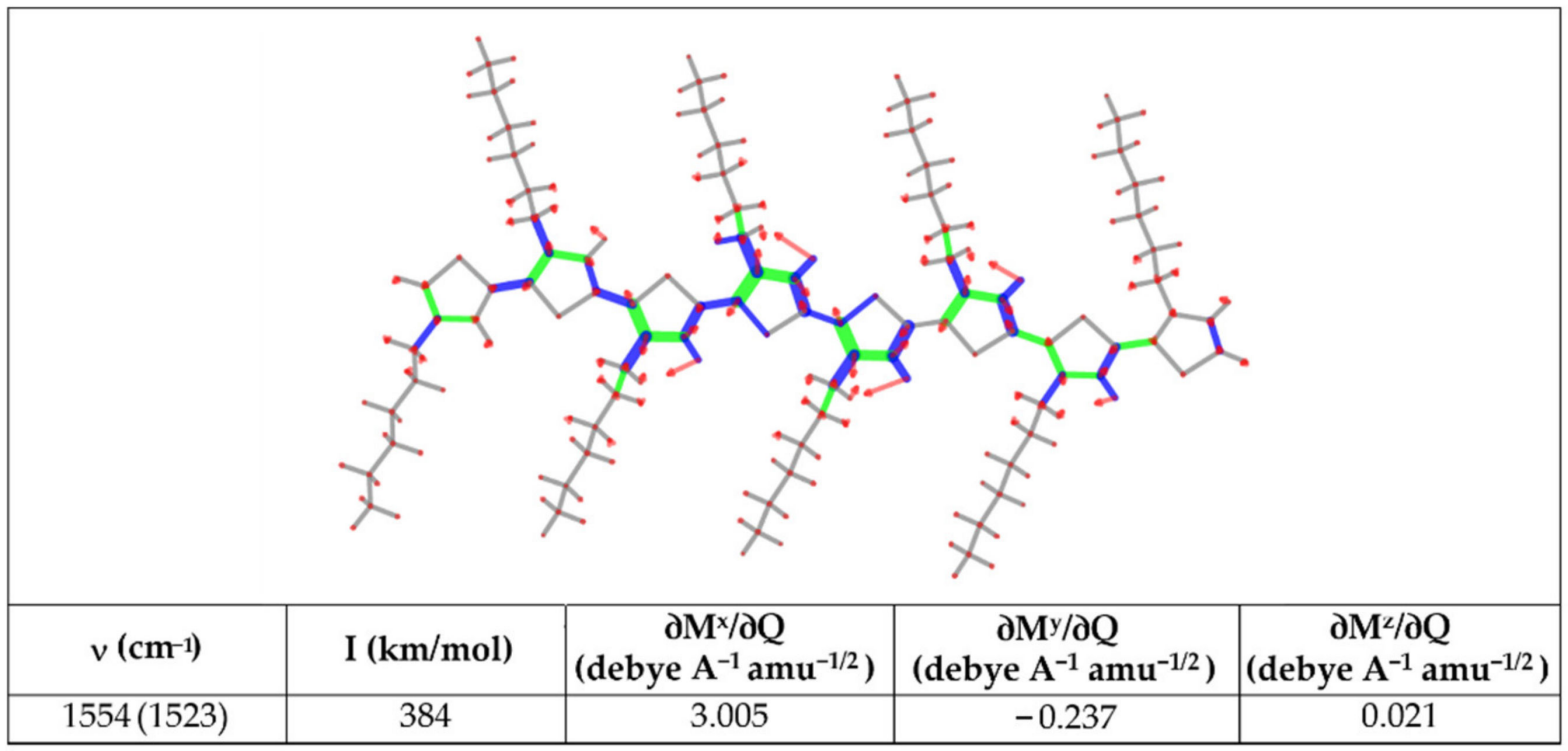
Computed vibrational displacements associated with the antisymmetric C=C stretching mode of a 3H8T molecule. This mode corresponds to the IR band observed at 1510 cm^−1^ (same wavenumber for the 3H8T oligomer and the P3HT polymer, see reference [63]). Computed values of the unscaled and scaled (in parenthesis) vibrational wavenumber, IR intensity, and associated dipole derivative vector are reported. Calculations have been carried out with density functional theory (DFT) simulations at B3LYP/6-31G(d,p) level; details in reference [63]. Red arrows represent displacement vectors; CC bonds are represented as green and blue lines of different thicknesses according to their relative stretching (shrinking).

Before adopting the above hypothesis for the rationalisation of the dichroism of the A band, we tested its robustness considering quantum chemical predictions carried out on 3H8T. Figure 5 shows the vibrational displacements (from the DFT computed vibrational eigenvector) of the normal mode associated with band A. The computed normal mode can be described as a collective R-, coupled with other vibrational displacements, namely, the stretching of the quasi-single CC bonds of the rings and of the C–C bond linking the alkyl chain to the ring, together with a small CH_2_ and CH wagging contribution. In spite of the complexity of the A normal mode compared to the R- vibration of the bare thiophene unit, the computed parameters reported in Figure 5 show that the dipole derivative exhibits a largely dominant component along the *x* direction, being the *x* direction of 3H8T one of the three inertia axes of the molecule, which practically coincides with the chain axis.

We can now confidently assume that the intensity of the band A is essentially produced by the component ∂M^x^/∂Q_A_, and we immediately realised that a good alignment of the P3HT parallel to the fibre axis should result in a parallel band, as indeed was observed. We measured the dichroic ratio of the A band (Table 2), thus obtaining an estimate of the degree of alignment of the P3HT chains by means of the Hermans function f. The parameter f can be translated into the Φ value, which describes an average tilting of the molecular chain axis, with respect to the fibre axis [81], of about 30°, both in the P3HT and in P3HT:PEO fibres. Since our result does not take into account the fact that the macroscopic alignment of the electrospun fibres along the direction of the rotating collector is not perfect, this finding points out that Φ is underestimated, and the P3HT chains are highly oriented along the fibre axis. Dichroism is evident both in spun P3HT:PEO and in P3HT fibres, thus demonstrating that the PEO removal by the fibre washing procedure does not cause appreciable rearrangements of the direction of the chain.

#### 3.2.3. IR Spectra of Iodine-Doped P3HT Nanofibres

**Figure 6:**
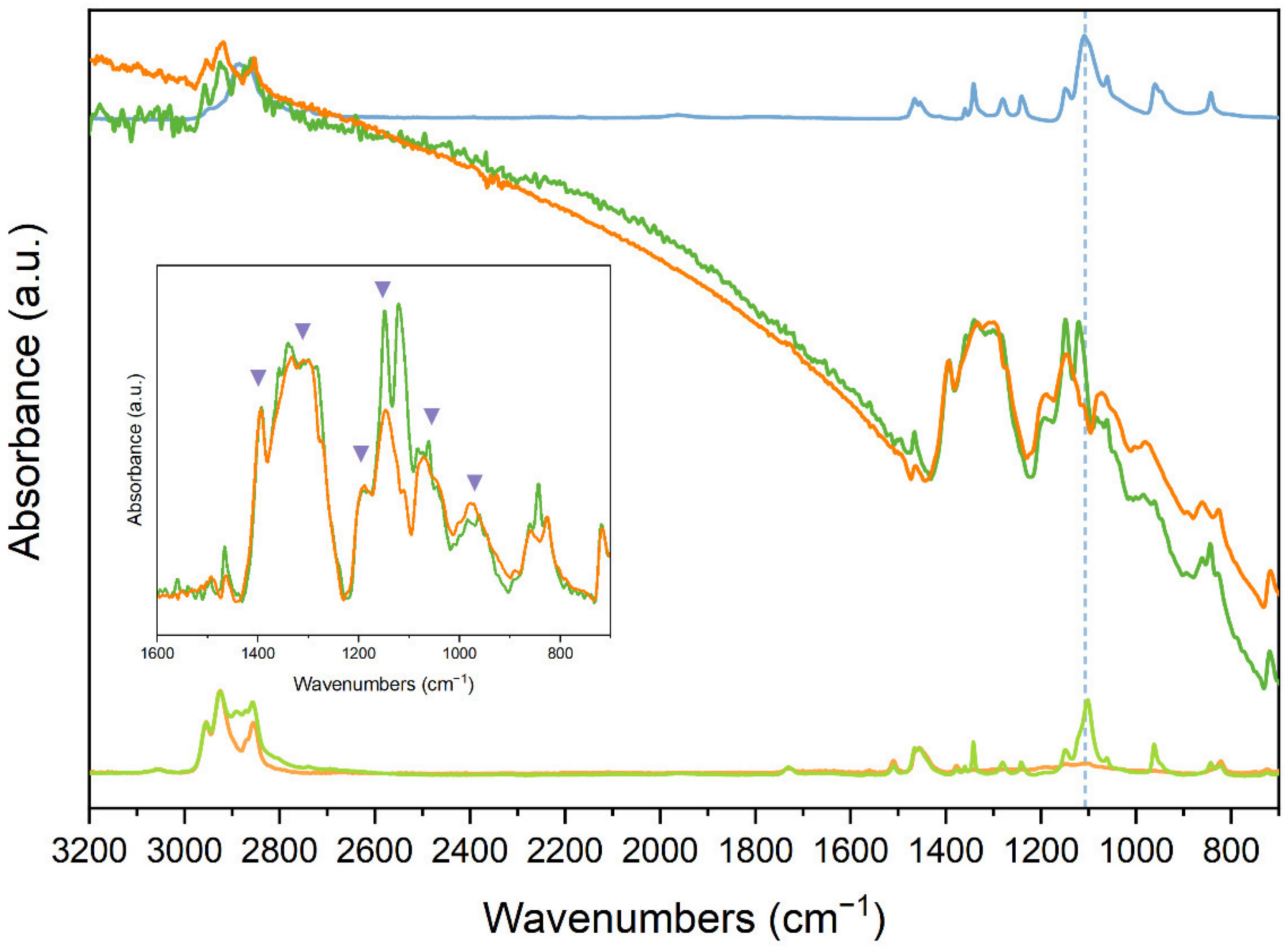
Infrared spectra of iodine-doped nanofibres of P3HT (**dark orange line**) and P3HT:PEO (**dark green line**); spectra have been normalised to the 1393 cm^−1^ peak. The zoom inset in the IRAV bands region shows the main peaks highlighted by violet triangles. As a reference, the IR spectrum of nanofibres of pure PEO (**blue line**), of the pristine P3HT (**light orange line**), and of pristine P3HT:PEO (**light green line**) are shown.

Figure 6 shows the appearance of the strong IRAV bands in heavily doped P3HT and P3HT:PEO. The wavenumbers of the main IRAV peaks are also reported in Figure 6, showing that there is a close correspondence of the IRAV features we observe (peaks at 1394; 1320; 1192; 1145; 1073; 976 cm^−1^) with data from the literature concerning chemically doped or electrochemically doped P3HT films [35,79]. As often observed, the IRAV pattern depends only slightly on the dopant or on the doping mechanism [42,44,69]. This is further evidence that IRAVs come from vibrational transitions associated with normal modes of the polarons, namely, to the vibrations of the chain segments that carry the charged defect, and they do not involve the dopant.

Looking at Figure 6, the strong intensity of the IRAV bands can be appreciated. The broad IRAV features show absorption intensities largely exceeding those of the CH stretching bands—the strongest absorptions in the IR spectrum of the pristine P3HT—which are clearly detectable in the 2800–3000 cm^−1^ region of the spectra of the doped samples. Incidentally, alkyl chains are unaffected by the charge injection by doping, thus, these bands can be used as an internal reference for a quantitative estimate of the IRAV intensities, which in turn depends on the concentration of doping-induced defects.

The data reported in Table 3 provide quantifications: at the highest doping regime we reached, the first structured IRAV feature in the range 1500–1300 cm^−1^ shows an increase in the absorption intensity, compared to the pristine state, of about 28 times in the case of P3HT fibres, and of about 13 times in the case P3HT:PEO. In the (unrealistic) hypothesis that all the thiophene monomeric units were affected by the doping, the above estimates mean that C-C bond stretching of the doped units carry IR intensities at least one order of magnitude higher than in the neutral case. We conclude that the absolute IRAV intensities (normalised to one thiophene ring, belonging to the charge defect) largely exceed, by a factor significantly larger than 10, the characteristic ring stretching intensities of the undoped thiophene units.

Although the iodine concentration diffused in the sample cannot be quantified, hence the actual polarons concentration, IR intensities ratios are very useful to monitor the evolution of the doping degree, as it will be illustrated in Section 3.2.4.

**Figure 7:**
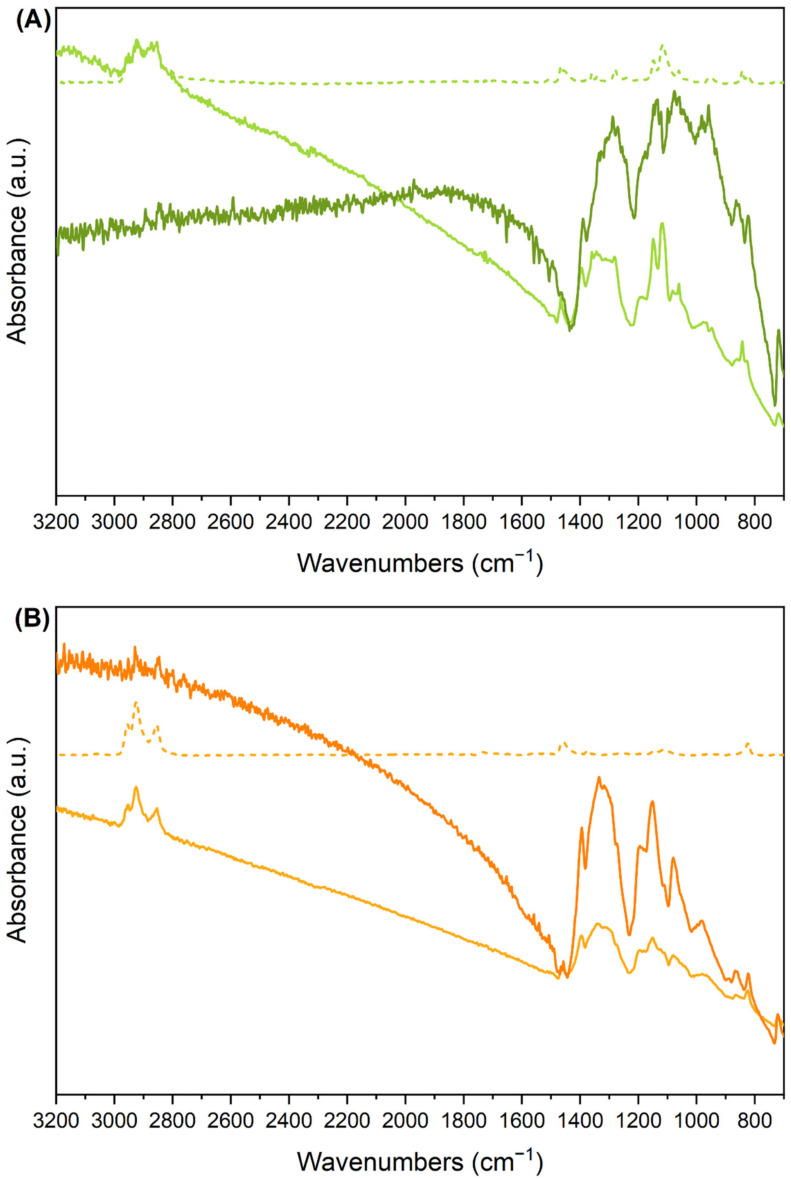
Infrared spectra of iodine-doped macroscopically aligned nanofibres recorded in polarised light. Panel (**A**), spectra of P3HT:PEO nanofibres; Panel (**B**), spectra of P3HT nanofibres. The darker lines refer to spectra recorded with light polarised parallel to the main axis of the nanofibres; lighter lines refer to spectra recorded with perpendicular polarisation. As a reference, dashed spectra of the pristine material recorded with perpendicular polarisation are reported.

The effective orientation of P3HT chains in the fibres allows one to obtain experimental information about the polarisation of the IRAV bands. We recorded spectra with polarised light parallel and perpendicular to the fibre axis (see Figure 7), showing a remarkable dichroism, which indicates that the transition dipoles associated with IRAVs are mainly directed along the polymer axis, in agreement with [35]. The key for the rationalisation of such behaviour, shared by all the IRAV bands, is their ECC character (see Section 3.2.1). In the case of doped polyenes and polyacetylene, the vibrational modes involving the BLA oscillation of a charged defect, namely, the ECC vibrations of the polarons, induce a large charge flux along the CC backbone. This results in a large dipole fluctuation along the chain axis direction (large ∂M^x^/∂Q_IRAV_), while exciting normal modes coupled with ECC. The mechanism has been proposed in the past based on empirical findings [42], and it has been confirmed by means of DFT modelling [74]. A similar explanation is applied to the present case.

#### 3.2.4. Monitoring of the De-Doping Process

Doping of conjugated polymers is a complex and inhomogeneous process, where the doping ratio is difficult to estimate, since it does not correspond to the nominal amount of doping agent used. Iodine is known to be highly diffusive in polymer chains, leading to relatively high doping ratios, with a clean process (sublimation rather than solution doping). On the other hand, iodide dopants tend to be released over time, especially when polymers with a relatively high ionisation energy are considered.

This specific issue, however, provides the opportunity to enable IR spectroscopy to monitor de-doping over time, namely, to assess the (in)stability of the doped state. Indeed, the measure of the intensity of the IRAV bands allows obtaining the trend of the de-doping processes with increasing time. According to the Lambert–Beer law, the absorption intensity of an IR band is proportional to the concentration of the chemical species to which the transition is ascribed. The linear relationship holds in the hypothesis that the transition dipole associated with the IR band is independent on the concentration of such chemical species.

**Figure 8:**
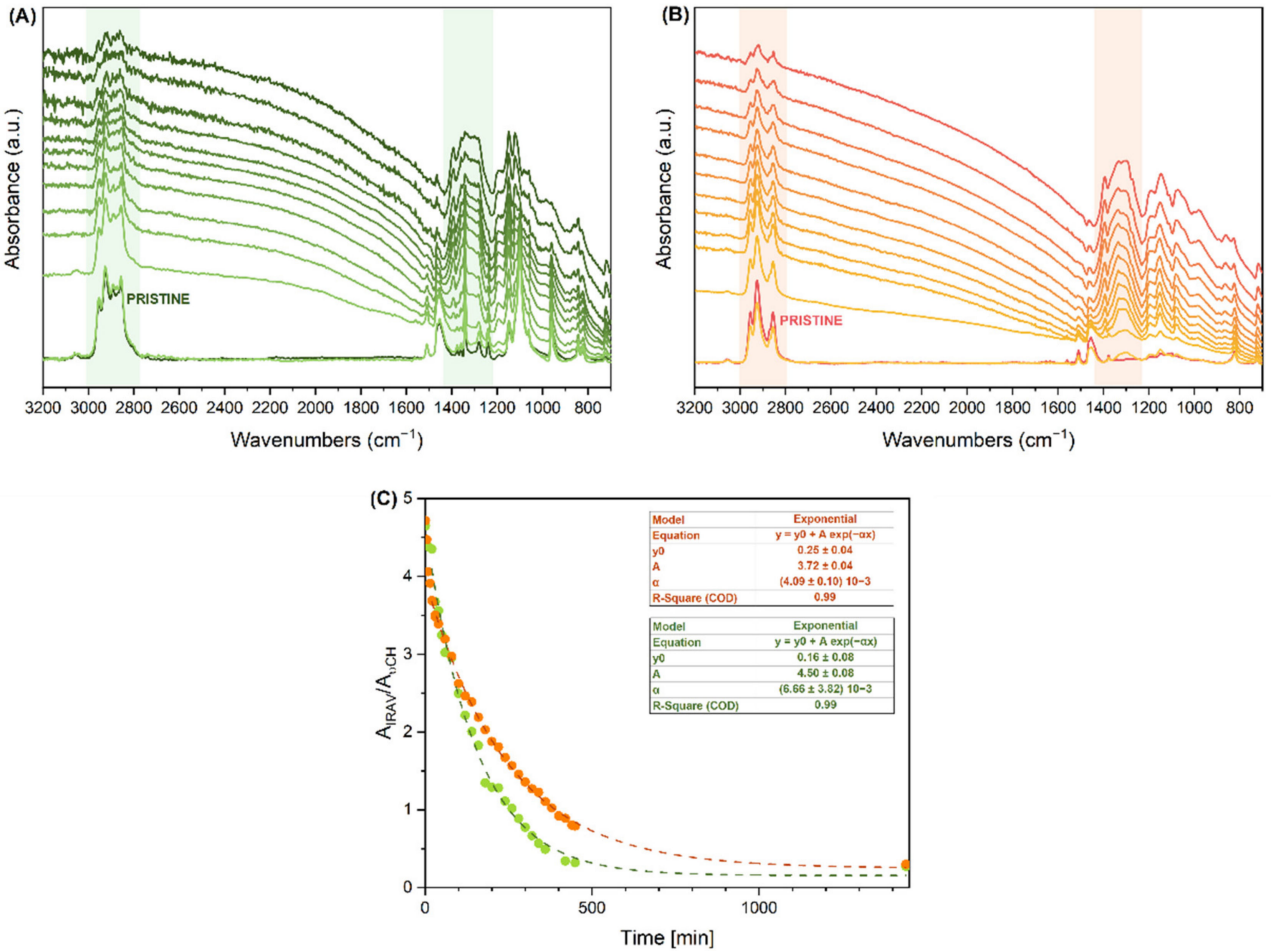
Evolution over time of the infrared spectrum of P3HT:PEO (green lines, panel (**A**)) and P3HT (orange lines, panel (**B**)) nanofibres doped with iodine vapours. A colour scale—from darker to lighter—has been adopted for plotting the spectra from the highly doped sample to those showing negligible IRAV intensities. Panel (**C**) shows the trend, over time, of the ratio among the area of the bands highlighted, namely A_IRAV_/A_CH_: the green dots refer to P3HT:PEO spectra—the contribution of CH stretching transition ascribed to PEO has been removed (see text); orange dots refer to P3HT spectra.

Looking towards the evolution of the IR spectra of the doped P3HT fibres (Figure 8), we can verify whether this hypothesis is reasonable for the IRAV bands. No changes in the peak frequencies or band shapes of the IRAV features are observed, while the decrease in intensities is clearly ascribed to the disappearance of the polarons after spontaneous de-doping. In other words, we do not have any evidence that the doping regime, which in the present case can be described as the polaronic regime at the initial doping level, changes over time in our samples. For this reason, the charge defects responsible for the IRAVs do not change during our observations, and thus present the same transition dipole. In conclusion, we can safely state that the intensity trend with time describes the trend of the polaron concentration.

Figure 8 shows the evolution of the IR spectra of highly doped P3HT and P3HT:PEO, from time t = 0 (immediately after the doping) to time t = 450 min, and then after one day. The sequence of spectra in Figure 8 shows a steep decrease in the intensity of the IRAV bands during the first six hours, for both samples. The plots in panel (C) were obtained by measuring the ratio between the area of the first IRAV bands (integrated intensity in the 1430–1220 cm^−1^ region) and the area of the whole CH stretching band (3000–2800 cm^−1^ region). For a correct comparison between the two samples, it is necessary to subtract the contribution by the PEO absorption in the lower wavenumber side of the CH stretching band. The procedure, which requires estimating the relative contribution from P3HT chains and PEO to the whole CH stretching integrated area, is illustrated in the Supplementary Materials.

The two plots show an exponentially decreasing trend for the two samples, and a very small residue of doping-induced features after one day. Interestingly, P3HT:PEO shows a steeper decreasing law, which indicates that the PEO scaffold does not function as a dopant diffusion barrier during the doping process, nor does it function in the de-doping process. This is consistent with the phase separation hypothesised by looking at the morphology before and after the PEO removal. The fact that doping is better retained in pure P3HT fibres with respect to the P3HT:PEO fibres can be ascribed to a rearrangement of the P3HT polymer chains, which occurs during the process of rinsing in acetonitrile, leading to well-packed polymer chains that better intercalate the dopants.

Some additional investigation concerning the P3HT morphology in the two P3HT:PEO and P3HT samples could contribute to the assessment of the above hypothesis. The analysis is grounded on the deconvolution of the dominant ECC Raman band, which possesses three different band components, associated with different P3HT phases [82]. The relative intensities (band areas) of the three components allow estimating the relative amount of: (i) the crystalline phase, (ii) a partially ordered phase, and (iii) a fully amorphous P3HT phase. The method has been illustrated in a detailed study of the morphology of regioregular P3HT samples and P3HT samples with different controlled levels of regiorandom sequences [82]; the paper demonstrates the very good agreement between the results from the Raman analysis and the DSC diagnosis reported in reference [83].

We have analysed the FT-Raman spectra of our pristine fibres, before and after PEO removal with acetonitrile, in order to put in evidence a possible evolution of the morphology (Figure S3). As already observed for several P3HT samples [82], both fibres show two overlapped major components of the ECC band, peaking, respectively, at 1445 and 1450 cm^−1^: these components have been associated with hairy A and hairy B phases, respectively [82]. The hairy A phase consists of 3D crystal domains in the crystal Form I [84], where P3HT chains in regular conformation (planar backbone formed by a sequence of coplanar thiophene units) are packed according to the crystalline order. The hairy B phase consists of chains with a quasi-planar backbone that do not show ordered 3D packing. A weak third component (1477 cm^−1^) is assigned to a fully amorphous phase, characterised by a large conformational disorder along the polythiophene backbone. Indeed, this component is the dominant one for P3HT samples in solution and in solid samples of regiorandom P3HT. Figure S3 illustrates the results of the Raman band deconvolution analysis, while the relative amounts of the three phases in the two fibre samples are reported in Table 4, together with the values obtained for the as-received P3HT powder.

The data reported in Table 4 show that, after washing the P3HT:PEO fibres, some rearrangement of the P3HT chains occurs, which determines an increase in the amount of the crystalline domains (hairy A phase). Interestingly, the phases composition of the washed fibres is very close to that shown by the P3HT powder. This finding confirms that the doping of the washed fibres is more stable because of a better packing of the P3HT chains compared to P3HT:PEO sample.

A further validation of the above conclusion comes from the UV-Vis absorption spectra of the two pristine samples. They show that in the washed fibres the absorption maximum is shifted at higher wavelength, with respect to the P3HT:PEO blend, due to an increase in crystallinity [85] (see Supplementary Materials, Figure S4).

#### 3.2.5. Raman Spectra of Pristine and Doped Fibres

**Figure 9:**
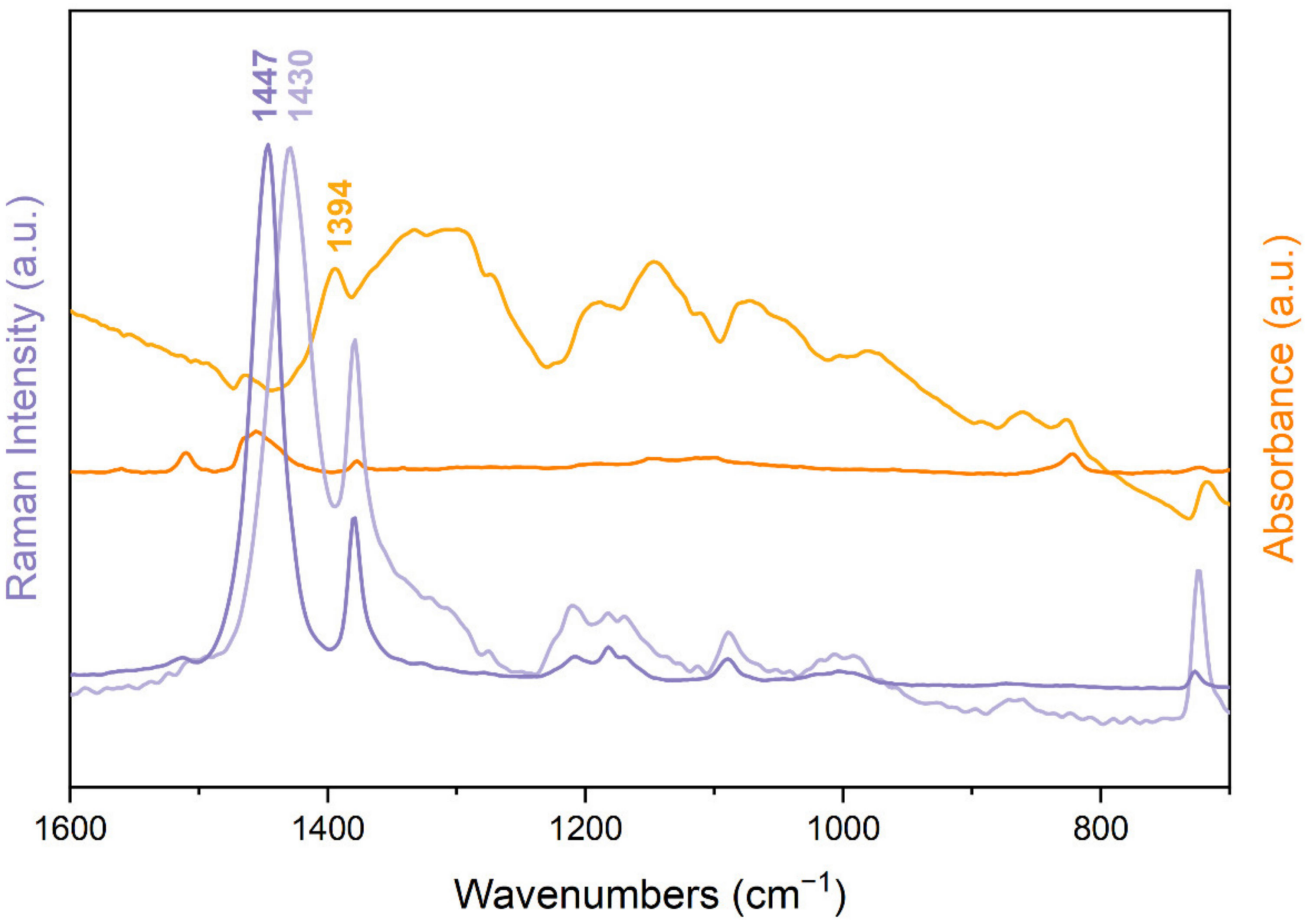
Violet lines are FT-Raman spectra of P3HT nanofibres. Orange lines are IR spectra of P3HT nanofibres. In both cases, the darker line refers to the pristine sample, the lighter line refers to the iodine-doped sample.

Independent proof of P3HT doping can be obtained through the analysis of the Raman spectrum. It is known that the Raman response of the polaron can be enhanced using an excitation laser line in resonance or near to the resonance with its electronic transition [70,86]. This indeed occurs with λ_exc_ = 1064 nm, as the spectrum reported in Figure 9 evidences. The doped material shows a very strong Raman transition at 1430 cm^−1^, which is ascribed to the Raman active ECC mode of the defected chains (RaAV), showing a downward shift of 17 cm^−1^ compared to the ECC line observed in the pristine case. The lower intensity Raman bands of the pristine and of the doped polymer, below 1250 cm^−1^, show minor differences.

Our observations parallel the evidence reported in references [70,86], for electrochemically doped P3HT. The comparison of Raman spectra of our samples with the spectra obtained by Lefrant et al. [70,86] allows us to state that, in the doping regime, we reached only the polaron formation stage. Indeed, the injection of two holes would determine a clear broadening and downward shift of the Raman band of the doped sample [70,86], because of the appearance of the ECC Raman transition associated with the bipolaron at lower wavenumber.

Interestingly, the direct comparison of the Raman spectrum and of the IR spectrum of the doped P3HT fibres shows that the strongest higher frequency IRAV feature is shifted downward in comparison of the main Raman active ECC main band both of the pristine and of the doped case. Indeed, the higher wavenumber IRAV peak occurs at 1394 cm^−1^, 36 cm^−1^ below the ECC Raman feature of the polaron. In addition, there is an impressive correspondence between the IRAV bands below 1250 cm^−1^ and the medium-/low-intensity Raman features of pristine and doped P3HT observed in the same spectra region.

The normal modes with wavenumbers below 1250 cm^−1^ are complex collective vibrations, which involve both CC stretching of the polythiophene chain and stretching and bending vibrations of the alkyl chains [63,64]. While their activation as IRAV can be explained by a non-vanishing contribution by the ECC vibration, the change in the CC stretching force constants of the defected region has a negligible effect in the modulation of the frequency of these normal modes, showing a dominant contribution from the vibrations of atoms scarcely affected by the charge transfer. On the contrary, the modulation in frequency of the main Raman band of the doped P3HT is another evidence of the large ECC content in its vibrational eigenvector.

In this framework, it may appear surprising that we observed a further downward shift in the higher frequency IRAV band. This behaviour indicates that the IR active and Raman active ECC modes are not the same. This has been demonstrated, by means of DFT calculations, in the case of a charge defect mimicking the charged polaron of polyacetylene [74]: because of the inversion centre of symmetry, the polaron shows a “gerade” Raman active ECC mode (RaAV) and an “ungerade” IR active ECC mode (IRAV). Moreover, this finding parallels the DFT results illustrated in reference [50], which discusses IR and Raman spectra of more complex p-doped polymers.

A detailed vibrational assignment of the IRAV and RaAV modes of P3HT, as well as the rationalisation of their intensities, certainly deserves further analysis based on state-of-the-art theoretical modelling of the charge defects and their vibrational dynamics.

## 4. Conclusions

This paper demonstrates that vibrational spectroscopy is an effective method to investigate the structural characteristics of pristine and doped P3HT in the peculiar case of highly anisotropic 1D structures.

The main achievements of the work are the following:

1. We show that it is possible to obtain defect-free and highly homogeneous fibres by electrospinning of a relatively high-molecular-weight P3HT in combination with PEO, which supports fibre formation. The scaffold polymer can be further removed by washing the fibres with acetonitrile without causing any remarkable change in the fibre appearance except for the expected size reduction.
2. We obtained P3HT:PEO and (washed) P3HT well-aligned fibres, by collecting the fibres onto a rotating drum.
3. All the fibres show marked IR dichroism, thus proving an effective molecular orientation of both PEO and P3HT, with the polymer chain axis mostly aligned parallel to the fibre axis.
4. The exposure to iodine vapours allows the effective doping of both P3HT and P3HT:PEO fibres, as demonstrated by IR spectra, with very strong IRAV features, typical of doped P3HT.
5. We set up a method, based on the measure of the intensity ratio between IRAVs and CH stretching absorptions, that allows the estimation of doping degree and enables the quantitative monitoring of the de-doping trends over time. These trends show that the de-doping process for both P3HT and P3HT:PEO fibres obeys an exponential decreasing law, with more rapid kinetics in the case of the P3HT:PEO. This evidence suggests that the washing procedure induces some refinement of the P3HT chain packing, which helps to stabilise the dopant–polymer complexes.
6. We obtained good Raman spectra of the fibre samples, both in the pristine and in the doped case. The clearly recognizable shift of the ECC band of the doped samples (RaAV), which is intensified by near to resonance excitation with a laser with a wavelength of 1064 nm, suggests that the Raman spectroscopy is a very effective probe, alternative to IR, for the determination of polaron formation.

Concerning possible technological applications, the above observations indicate that micro/nanofibres of P3HT can be obtained by the electrospinning procedure, opening the way to the production of high-surface-area membranes, which could find application in the sensing of analytes able to dope the polymer. Importantly, P3HT can be doped even in the presence of a scaffold polymer (i.e., PEO), which improves the mechanical characteristics of the membrane. Moreover, electrospinning is very suitable for tuning the fibre structure (e.g., the fibre’s average diameters) by means of the selection of the process parameters. This offers the opportunity to modify and control several physical properties of the material. In this framework, the present contribution represents the initial, promising step, which will allow the design of new experiments focusing on the optimisation of conducting organic fibres. In particular, in addition to the fine tuning of the fibre size, strategies for the refinement of the morphology, e.g., annealing processes, should be considered. Worth noting is that the fundamental characterisation method we developed enables a fast and reliable analysis of the doping of conducting polymer fibres, thus contributing to the selection of polymer/dopant pairs for highly conductive organic materials. Hence, IR and Raman spectroscopies are eligible techniques to monitor the charge carrier in doped polymers, as complementary or alternative to electrical tests.

Notably, the availability of oriented doped samples shines light on the fundamental properties of the vibrational spectra of polarons, which show markedly parallel polarised IRAVs. This information suggests that large electron charge fluxes along the polymer chain take place during polaron vibration, which in turn experimentally proves the existence of a phonon-assisted intrachain charge hopping mechanism in doped P3HT.

The approach illustrated in this paper can be extended to the structural characterisation of several pristine and doped polymers, both as nanofibres and as films. This opens up new perspectives in the development of structure/properties relationships toward the optimisation of conducting organic materials, e.g., experimenting with different polyconjugated polymers and different dopant molecules.

## Figures and Tables

**Table 1:** Wavenumber of the IR marker bands of P3HT and their vibrational assignment [63,64].

	ν (cm^−1^)	Vibrational Assignment
A	1510	Antisymmetric C=C str of thiophene ring C–C str (alkyl chain); CH wagging
B	1455–1465	ECC mode–CH_2_ bending
C	1377	CH_3_ umbrella motion
D	823	Thiophene ring CH out-of-plane bending; CH_2_ rocking

**Table 2:** Observed IR dichroic ratio for the IR band A (antisymmetric C=C stretching) of P3HT in the two macroscopically oriented fibres of P3HT and of the blend P3H:PEO. The Hermans function f and the average tilting angle (Φ) provide an estimate of the degree of molecular orientation of the P3HT chains along the fibre axis.

	R = A_//_/A_⊥_	f	Φ (deg)
P3HT	5.93	0.62	30.14
PEO:P3HT	5.80	0.61	30.43

**Table 3:** Ratios of the integrated band intensities in the CC stretching region (1430–1220 cm^−1^) and of the whole CH stretching intensity (3000–2800 cm^−1^) for pristine and doped samples.

	P3HT	P3HT:PEO
	R = A_1430−1220_/A_3000−2800_	R = A_1430−1220_/A_3000−2800_
Pristine	0.169	0.227
Highly doped	4.715	2.899
[R_doped_/R_pristine_]	28	13

**Table 4:** Relative amount of the hairy A, hairy B, and fully amorphous phases for the P3HT fibres under investigation. The data are obtained from the intensity ratios of the three band components resulting from the deconvolution of the ECC Raman band. Values obtained for the P3HT powder are reported for sake of comparison.

	P3HT	P3HT:PEO	P3HT
	powder	fibres	washed fibres
Hairy A	54%	44%	53%
Hairy B	45%	53%	46%
Fully amorphous	1%	3%	1%

## Data Availability

Data is contained within the article or Supplementary Material.

## Supplementary Materials

The following supporting information can be downloaded at https://www.mdpi.com/article/10.3390/nano12234308/s1. Figure S1: SEM micrographs of P3HT:PEO nanofibres from low-Mw P3HT (21,000 g/mol); Figure S2: SEM micrographs of P3HT:PEO nanofibres (P3HT Mw = 50,000–75,000 g/mol) and diameter distribution of P3HT samples; Figure S3: FT Raman spectra deconvolution (Fityk 0.9.8 software) in the region 1300–1600 cm^−1^; Figure S4: UV-Vis spectra of P3HT:PEO and P3HT washed nanofibres. Description of the procedure for the removal of the PEO CH stretching contribution from P3HT:PEO samples.
