# Supplementary material for: Conducting Electrospun Nanofibres: Monitoring of Iodine Doping of P3HT through Infrared (IRAV) and Raman (RaAV) Polaron Spectroscopic Features
Source: Nanomaterials (Basel). 2022 Dec 4;12(23):4308. doi: 10.3390/nano12234308 (PMC9739408; doi:10.3390/nano12234308)
Supplement: Supplementary file 1 [file nanomaterials-12-04308-s001.zip › nanomaterials-2045332-supplementary.pdf]

Conducting Electrospun Nanofibres: Monitoring of Iodine Doping of P3HT through Infrared (IRAV) and Raman (RaAV) Polaron Spectroscopic Features

Alessia Arrigoni [1], Luigi Brambilla [1], Chiara Castiglioni [1,*] and Chiara Bertarelli [1,2]

[1] Dipartimento di Chimica, Materiali e Ingegneria Chimica Giulio Natta, Politecnico di Milano, Piazza Leonardo da Vinci 32, 20133 Milan, Italy
[2] Center for Nano Science and Technology @PoliMi, Istituto Italiano di Tecnologia, Via Pascoli 70/3, 20133 Milan, Italy
* Correspondence: chiara.castiglioni@polimi.it

SUPPORTING INFORMATION

Doping quantification from IR bands intensities.

Procedure for the removal of the PEO CH stretching contribution from P3HT:PEO samples.

$$I_{\nu_{CH}}^{TOT} = I_{\nu_{CH}}^{PEO} + I_{\nu_{CH}}^{P3HT}$$

$$\begin{cases} \dfrac{I_{\nu_{CH}}^{P3HT}}{I_{R^-}^{P3HT}} = A \\ \dfrac{I_{\nu_{CH}}^{TOT}}{I_{R^-}^{P3HT:PEO}} = B \end{cases} \rightarrow \frac{I_{\nu_{CH}}^{P3HT}}{I_{\nu_{CH}}^{TOT}} = \frac{A}{B} = x$$

Where x is the contribution of P3HT CH stretching in the CH stretching band in P3HT:PEO spectra.

Then, using:

$$I_{\nu_{CH}}^{P3HT} = x * I_{\nu_{CH}}^{TOT}$$

It is possible to obtain the contribution of $I_{\nu_{CH}}^{P3HT}$ with respect to the total intensity of CH stretching.

In our case,

$I_{\nu_{CH}}^{P3HT}$	$I_{R^-}^{P3HT}$	$I_{\nu_{CH}}^{TOT}$	$I_{R^-}^{P3HT:PEO}$	A	B	x
19.16	0.52	34.13	0.58	36.85	59.05	0.62

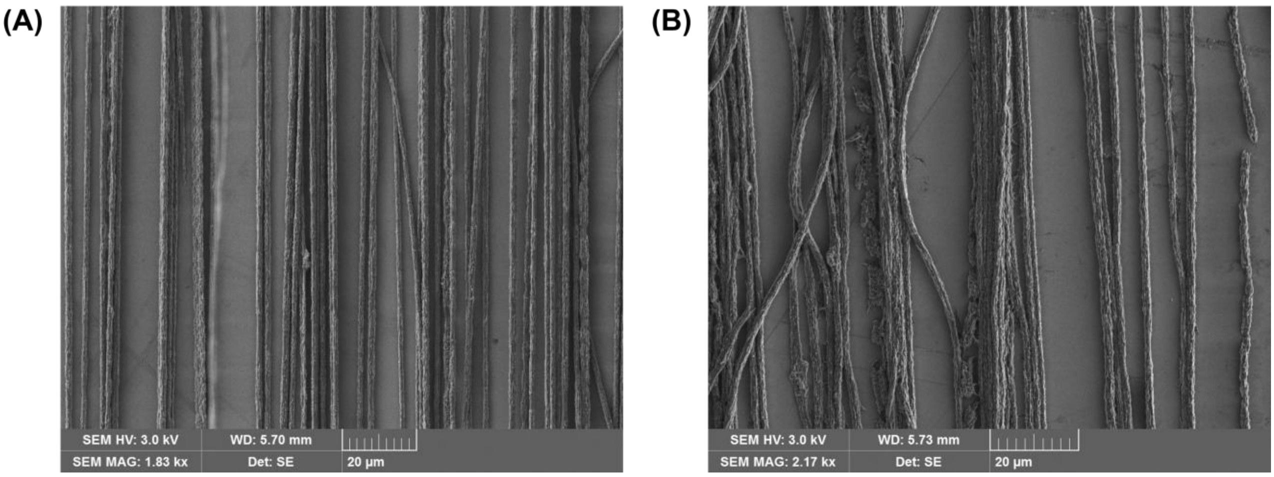

Figure S1: SEM micrographs of P3HT:PEO nanofibres from low Mw P3HT (21000 g/mol) panel (A) as deposited, panel (B)) P3HT samples after removal of PEO

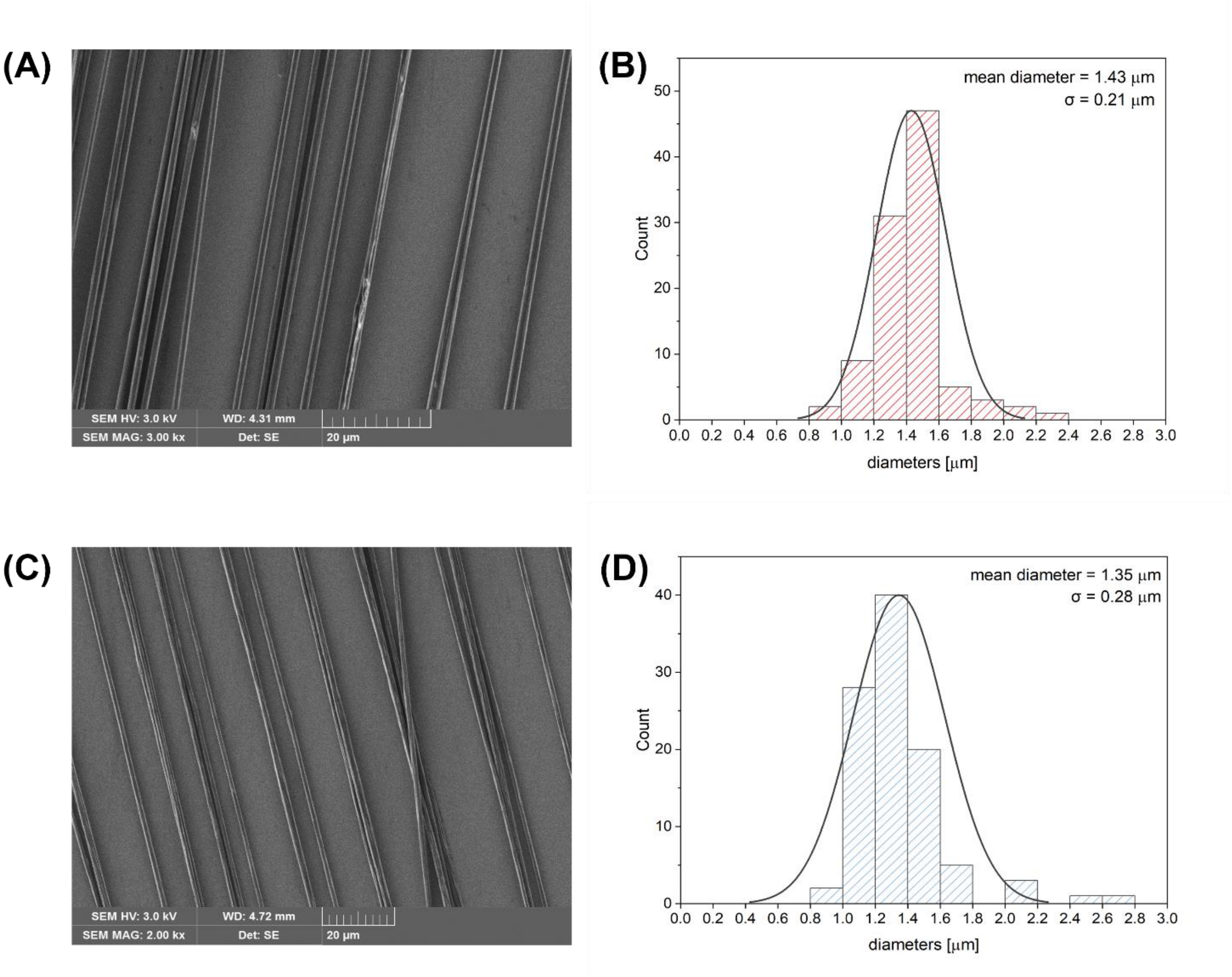

Figure S2: SEM micrographs of P3HT:PEO nanofibers (P3HT Mw = 50000-75000 g/mol): panel (A) as deposited, panel (B) diameters distribution of as deposited samples, panel (C) P3HT samples after removal of PEO, panel (D) diameters distribution of P3HT samples

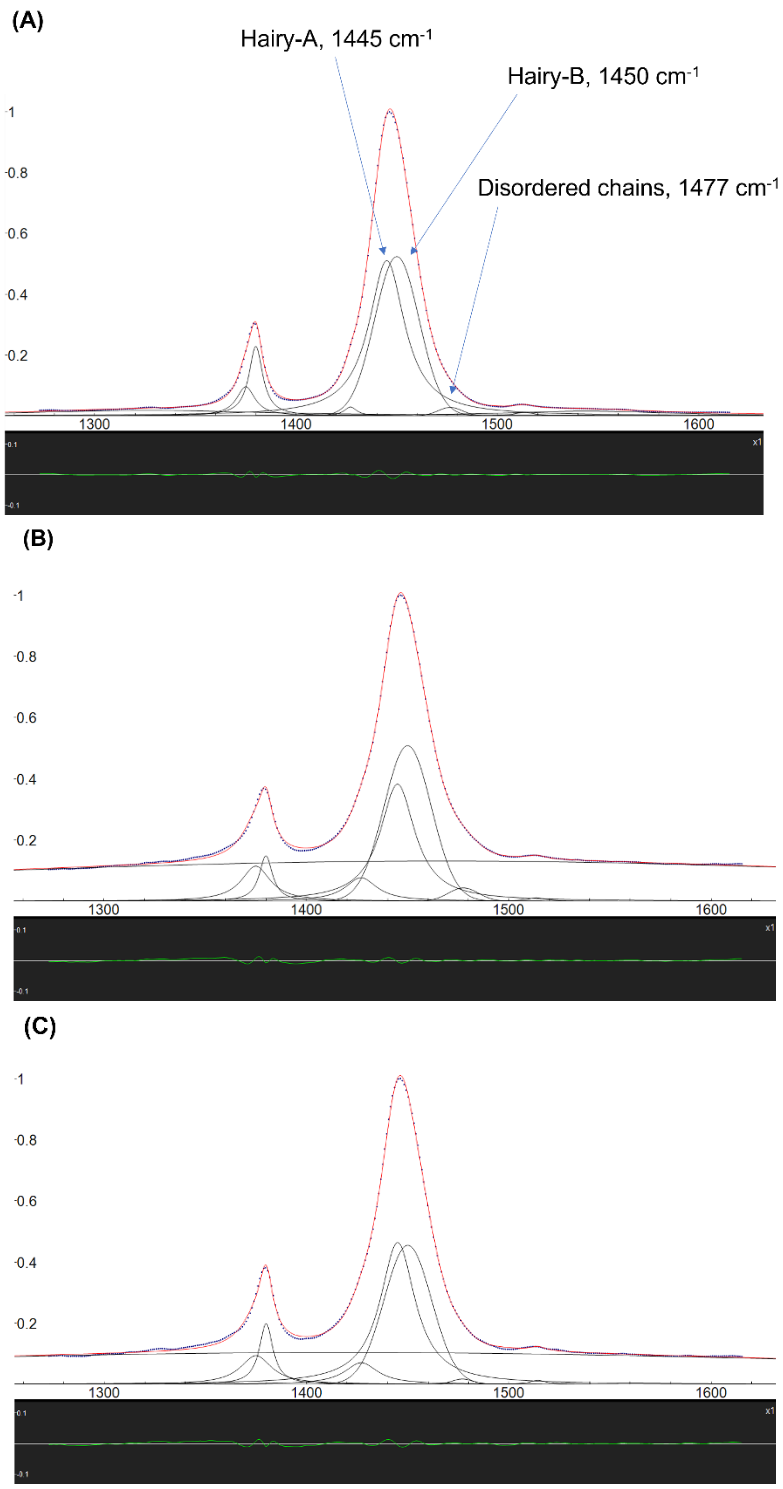

Figure S3: FT Raman spectra deconvolution (Fityk 0.9.8 software) in the region 1300 – 1600 cm^{-1}. The method proposed in ref [87] has been adopted. Panel (A) P3HT, powder; panel (B) P3HT:PEO nanofibers; panel (C) P3HT washed nanofibres.

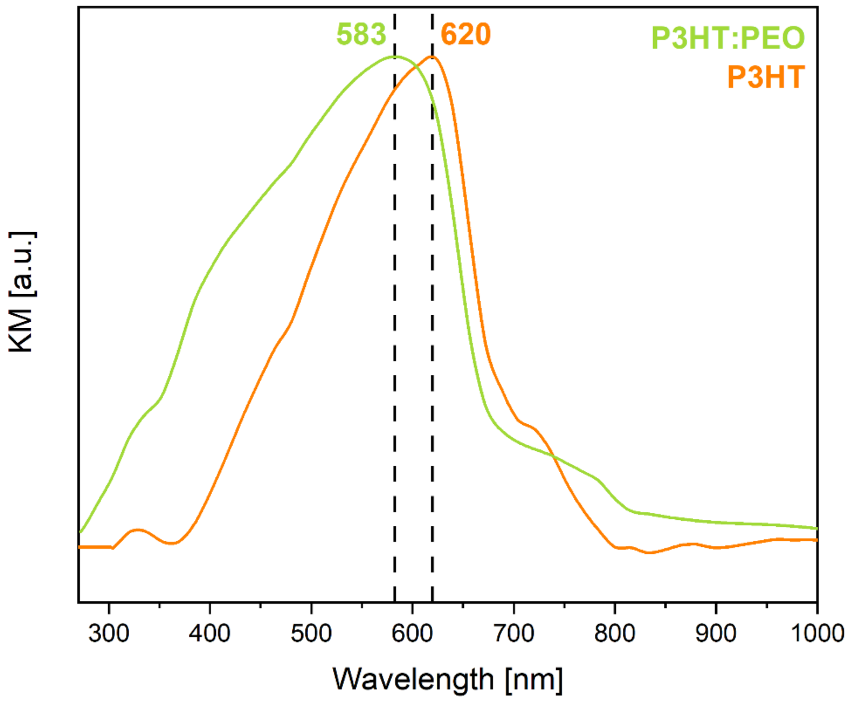

Figure S4: UV-Vis spectra of P3HT:PEO (green line) and P3HT washed (orange line) nanofibers. Spectra were recorded with a Jasco W-570 spectrophotometer in the range 250 – 1000 nm. Diffused reflectance set up with an integrating sphere has been adopted. The spectra are reported after Kubelka-Munk transformation.
